# The Impact of Intimate Partner Violence on Sexual Attitudes, Sexual Assertiveness, and Sexual Functioning in Men and Women

**DOI:** 10.3390/ijerph18020594

**Published:** 2021-01-12

**Authors:** Juan Carlos Sierra, Ana I. Arcos-Romero, Ana Álvarez-Muelas, Oscar Cervilla

**Affiliations:** Mind, Brain, and Behavior Research Center (CIMCYC), University of Granada, 18011 Granada, Spain; aiar@correo.ugr.es (A.I.A.-R.); alvarezm@ugr.es (A.Á.-M.); ocervilla@ugr.es (O.C.)

**Keywords:** intimate partner violence, psychosexual variables, sexual functioning

## Abstract

Background: Intimate Partner Violence (IPV) causes physical, sexual, or psychological harm. The association between psychosexual (sexual assertiveness, erotophilia, and attitude towards sexual fantasies) and sexual function (sexual desire, sexual excitation, erection, orgasm capacity, and sexual satisfaction), and the experience of physical and non-physical IPV was assessed. Methods: Data from 3394 (1766 women, 1628 men) heterosexual adults completed the Spanish version of the Index of Spouse Abuse, scales measuring psychosexual and sexual function, and demographic characteristics were collected. Results: For men, poorer sexual health was associated with an experience of physical abuse (F = 4.41, *p* < 0.001) and non-physical abuse (F = 4.35, *p* < 0.001). For women, poorer sexual health was associated with physical abuse (F = 13.38, *p* < 0.001) and non-physical abuse (F = 7.83, *p* < 0.001). Conclusion: The experience of physical or non-physical abuse has a negative association with psychosexual and sexual functioning in both men and women.

## 1. Introduction

Intimate Partner Violence (IPV) refers to behaviors that take place as part of partner relationships and cause physical, sexual, or psychological harm to victims of abuse, sexual coercion, psychological abuse, and controlling behaviors [1]. Non-physical or psychological IPV is the most prevalent type and involves insults, humiliation, and controlling behaviors, which inflicts serious psychological harm [2,3,4]. Sometimes, psychological IPV is manifested regardless of other types [3], such as physical or sexual IPV, and it can form a routine part of a relationship [5]. Psychological IPV often comes first and is an important risk factor for physical IPV [4,6]. It has been reported that perceived abuse severity and the perpetrator’s responsibility differ according to the type of abuse (non-physical or physical) [7], as well as the gender of the victim and the perpetrator aggressor [8]. Thus, physical abuse is considered to be more severe than non-physical abuse, and these associations hold whether it is male towards female, or female on male abuse [9]. It is essential to identify irrational beliefs and distorted thoughts among perpetrators and in the general population [10].

The experience of abuse differs between men and women. In Spain, up to 25% of the women who attend healthcare services have experienced, or presently experience, IPV, with greater prevalence in those from a lower socio-economic stratum and the more vulnerable [11]. Based on the 2019 Macro-Survey on Violence Against Women, 11% of Spanish women aged over 16 years have suffered physical abuse by their present or past partner at some time in their lives, while the prevalence of non-physical abuse ranges between 24.2% and 28% [12]. In the USA, the Partner and Sexual Violence Survey indicated that, in the relationship context, one in four women and one in seven men have experienced physical abuse, whereas almost half of the women and men had experienced at least one form of non-physical abuse [13]. Specifically, IPV is the most widespread form of violence experienced by women [14,15] and tends to start before 25 years of age [13], which means that it can be considered a global public health problem [16,17,18].

The consequences of IPV can be physical, social, and psychological [19,20,21,22]. They have been related to adverse effects in mental, relational (e.g., relationship satisfaction or attachment), and sexual (e.g., sexual satisfaction, dysfunction, sexual communication) well-being; the effect on women is more harmful [23]. Thus, IPV could influence sexual health [24]. Sexual assertiveness has been related to IPV, which is a predictor of less non-physical abuse, and it is associated with better identification of violence in partner relationships [25]. Non-physical abuse has been negatively associated with assertiveness to initiate and to refuse sexual activity in men [26,27] and women [26]. Considering the role that sexual attitudes have for sexual health, they also could be associated with abuse in partner relationships. Sexual attitudes are a learned disposition to respond to sexual stimuli that are associated with expectation and sexual behaviors [28]. Sexual attitudes can occur towards sexuality in general (i.e., erotophilia) or specific sexual behaviour (i.e., sexual fantasies). Erotophilia is considered an indicator of sexual health [29], and it is also related to better sexual functioning [30,31], sexual desire [32], subjective orgasm experience [33], and sexual assertiveness [26,34]. Furthermore, the positive attitude towards sexual fantasies has been positively related to sexual functioning [28,35,36], erotophilia [26,28,37], and sexual assertiveness to initiate sexual activity [26,28]. The attitude towards sexual fantasies has been negatively associated with assertiveness to refuse undesired sexual activities [28].

It suggests that non-physical abuse could have the ability to predict sexual functioning in women [38]. Furthermore, physical abuse experience implies a worsening in sexual functioning dimensions in women [39]. To have experienced abuse in a partner relationship has been related to sexual functioning dimensions. Physical IPV has been associated with a higher prevalence of risky sexual behavior, a higher risk of sexually transmitted diseases (STD), unplanned pregnancies, abortion, a higher probability of dyspareunia, and less sexual pleasure [40]. In addition, physical abuse could be a negative predictor of sexual desire [41]. Specifically, non-physical and physical IPV have been associated with less sexual satisfaction in women [42,43,44,45].

To provide evidence of the violence that takes place in partner relationships, and to determine the association between IPV and sexual health in the heterosexual population, the present study examines differences in sexual health variables in men and women who have suffered IPV from the general heterosexual population. The main objective is to compare psychosexual variables (i.e., sexual assertiveness, erotophilia, and positive attitude towards sexual fantasies) and sexual functioning variables (i.e., sexual desire, sexual excitation, orgasmic capacity, erection, and sexual satisfaction) in men and women who: (1) have suffered non-physical IPV vs. have not suffered non-physical IPV; (2) have suffered physical IPV vs. have not suffered physical IPV. We hypothesized that those who have experienced some type of partner abuse would experience poorer sexual function, less sexual assertiveness, and more negative sexual attitudes than those who had not experienced abuse.

## 2. Materials and Methods

### 2.1. Participants

We determined the sample size based on 97% confidence level and 3% error estimation. Considering the size of the total Spanish population, we estimated that we would need at least 1308 men and 1308 women. The sample was drawn from the general Spanish population. The inclusion criteria were Spanish nationality, being 18 years old or older, heterosexual orientation, and being currently involved in a heterosexual relationship. People with a same-sex partner were dismissed from these analyses.

### 2.2. Instruments

Background and Sexual History Questionnaire. This includes information about age, sex, nationality, education level, sexual orientation with items of the Kinsey Scale, partner’s relationship, and sexual activity (i.e., to have sexual relations within the partner’s relationship).

The Spanish version of Index of Spouse Abuse [46]. The version from Sierra et al. [47] was used for women. It is composed of 19 items distributed into two subscales: Non-physical abuse (Cronbach’s alpha = 0.93) and Physical abuse (Cronbach’s alpha = 0.89). The version from Santos-Iglesias et al. [27] was used for men. It is composed of 30 items distributed into three subscales: Non-physical abuse (Cronbach’s alpha = 0.81), Behavioral control (Cronbach’s alpha = 0.60), and Physical abuse (Cronbach’s alpha = 0.79). To have the same two dimensions of abuse for both men and women, the subscale titled Behavioral control was not considered in this study. The frequency of abuse is recorded on a Likert scale from 0 (never) to 4 (most of the time). Ordinal’s alpha coefficient was 0.90 for men and 0.93 for women in physical abuse. It was 0.95 for both sexes in non-physical abuse.

The Spanish version of Sexual Assertiveness Scale (SAS) [48,49]. It evaluates sexual assertiveness. The scale is composed of 18 items answered on a 5-point Likert scale from 0 (never) to 4 (always). The items are distributed into three dimensions: Initiation of sexual activity, Refusal of unwanted sexual contact, and STI-prevention (Sexually Transmitted Infections). Higher scores indicate greater sexual assertiveness. The scale has shown a stable factorial structure, Cronbach’s alpha coefficients ranged from 0.66 to 0.86 [49]. In the present study, ordinal alpha coefficients ranged between 0.67 and 0.91 for men, and between 0.78 and 0.93 for women.

The Spanish version of Sexual Opinion Survey (SOS-6) [34,50]. The scale evaluates the erotophilia. It consists of 6 items answered on a 7-point Likert scale from 1 (totally disagree) to 7 (totally agree). Higher scores indicate higher erotophilia. Its internal consistency is adequate, Cronbach’s alpha is 0.74 [34]. In the present study, ordinal alpha was 0.82 for both men and women.

The Spanish version of Hulbert Index of Sexual Fantasy (HISF) [28,51]. The scale evaluates a positive attitude towards sexual fantasies. It is composed of 10 items answered on a 5-point Likert scale from 0 (never) to 4 (always). Higher scores indicate a more positive attitude towards sexual fantasies. The internal consistency of the Spanish version estimated by ordinal alpha is 0.94 [28]. In the present study, ordinal alpha coefficient was 0.92 and 0.94 in men and women, respectively.

The Spanish version of Sexual Desire Inventory (SDI) [52,53]. It assesses interest in sexual activity. It is composed of 13 items distributed into three dimensions: Partner-focused dyadic sexual desire, Dyadic sexual desire for an attractive person, and Solitary sexual desire. Higher scores indicate greater sexual desire. Its Cronbach’s alpha coefficients range between 0.80 (Partner-focused dyadic sexual desire) and 0.90 (Solitary sexual desire) in men and between 0.89 (Dyadic sexual desire for an attractive person) and 0.93 (Solitary sexual desire) in women [52]. In the present study, Cronbach’s alpha ranged between 0.80 in men and 0.88 in women (Partner-focused dyadic sexual desire), between 0.87 in men and 0.89 in women (Dyadic sexual desire for an attractive person), and 0.90 in men and 0.92 in women (Solitary sexual desire).

The Spanish version of Massachusetts General Hospital Sexual Functioning Questionnaire (MGH-SFQ) [54,55]. It evaluates the general sexual functioning during the previous month in the following areas: sexual desire, arousal, orgasm, erection (only for men), and overall sexual satisfaction. It consists of five items answered on a 5-point Likert scale from 0 (totally absent) to 4 (normal). Higher scores indicate better sexual functioning. The internal consistency of the Spanish version is 0.90 and 0.93 in men and women, respectively [55]. In this study, ordinal alpha was 0.91 for men and 0.71 for women.

### 2.3. Procedure

The sample was selected using a non-probability quota sampling method by quotas according to sex and age. Participants were evaluated individually or in small groups at public places (e.g., universities, health centers, and social centers) by trained researchers who provided the instructions for participation. Snowball sampling was used. Those participants evaluated at university or social centers willing to collaborate were given one or two copies of the instruments and envelopes, according to their chances to pass them on to one or two people under the same conditions as them. The answered questionnaires were returned closed in an envelope, within a week. Firstly, the informed consent was accepted. Participants were given information about the objective, guaranteeing anonymity, confidentiality of responses, and data protection. No compensation for taking part in the study was received, it was voluntary. The time to complete it was approximately 20 min.

### 2.4. Analytic Sample and Strategy

We decided to only include those participants who had completed all the sociodemographic questions. For the sexual health scales, only those participants who had answered at least 75% of the items of each dimension were included. To control missing data, we counted values within cases for each examined dimension and calculated the percentages of missing data. Due to the low percentages found, we proceeded to replace missing values using the “median of nearby points” method with the total amplitude of the points. In the case of the IPV dimensions, we found the following percentages of missing data: 3% for non-physical abuse and 1.2% for physical abuse in men; 2.1% for non-physical abuse and 1.2% for physical abuse in women.

As recommended by Zumbo et al. [56], the internal consistency of the scales was estimated using the ordinal alpha coefficient, except for SDI for which Cronbach’s alpha was obtained because it includes response scales of more than six points. In order to compare variables of sexual health in men and women considering whether they have or not experienced abuse, we examined the association between an experience of abuse and sexual health dimensions while controlling for sociodemographic characteristics. First, the sociodemographic characteristics were compared by Student’s *t* and Chi-squared. The differences within each group were calculated by comparison of column proportions, adjusting *p* values for Bonferroni correction. To examine the relation with abuse experience, four separate multivariate analyses of variance while controlling covariates were conducted (MANCOVA). As Box’s *M* test of equality of covariance matrices was significant (*p* < 0.001) for all the multivariate tests, we interpreted Pillai’s trace value. Furthermore, to test significant differences between groups in each sexual health variable individually, we considered univariate *F*-tests (ANOVA) for the test of between-subjects’ effects. The effect size was estimated using partial eta squared [57]. The groups were incidentally created with a balanced proportion in each subsample according to sex (Men/Women), type of abuse (Non-physical/Physical), and having experienced abuse (Yes/No). Each type of abuse was the independent variable. The dependent variables were both the psychosexual dimensions (sexual assertiveness, erotophilia, and positive attitude towards sexual fantasies) and the sexual functioning dimensions (sexual desire, sexual excitation, orgasmic capacity, erection, and sexual satisfaction). Covariates were the sociodemographic variables that previously showed significant differences across groups (see Table 1). We decided to control them in order to reduce the magnitude of the error term. The final multivariate analyses were as following: (1) Men who reported or not non-physical abuse while controlling their education level; (2) Men who reported or not physical abuse while controlling their age; (3) Women who reported or not non-physical abuse while controlling their age and education level; and (4) Women who reported or not physical abuse while controlling their age, sexual activity, and education level.

## 3. Results

The estimated minimum number of participants was 1308 men and 1308 women. The rejection rate was low, less than 10% approximately. The primary sample consisted of 4034 participants (1901 men and 2133 women) with ages ranging between 17 and 87 years old. The final analytic sample of respondents who met inclusion criteria was 3394 (1628 men, 1766 women) and their ages ranged between 18 and 81 years old (M = 36.16; SD = 13.30). A total of 99.62% of the participants reported having sexual relations within their relationship. Regarding the education level, 52% reported university studies, 27.9% secondary studies, 18% primary studies, and 2.1% had not completed primary school.

From the total sample, we incidentally created groups according to the type of abuse and those who reported to have experienced abuse or not have experienced abuse. We calculated the total scores of the dimensions for Non-physical abuse and Physical abuse. Taking into account that these are two different variables, they included different items for men and women. We did not make comparisons between the Non-physical abuse group and the Physical abuse group. We used the scores from the Index of Spouse Abuse [46] to create the examined groups. Participants from the Abuse group were those who, in at least one item of the ISA, marked the option from 1 “rarely” to 4 “most of the time”; the Non-abuse group was made up of those participants whose scores were equal to 0 “never”. It was not controlled whether participants from the non-physical abuse group may also be part of the physical abuse group. The types of abuse have been analyzed independently. Table 1 presents the sociodemographic characteristics of the sample and the differences between groups.

In men, results showed a significant association between having experienced non-physical abuse and the sexual health variables while controlling education level. In Table 2, the univariate *F*-tests indicated that there were significant differences between having experienced non-physical abuse or not, in assertiveness to initiate sexual activity, assertiveness to refuse sexual contact, partner-focused dyadic sexual desire, general sexual desire, and general sexual satisfaction. Furthermore, to have experienced physical abuse was significantly associated with the sexual health variables when we controlled age in men.

In women, to have experienced non-physical abuse was significantly associated with the sexual health variables when controlling age and education level. In Table 3, the univariate *F*-tests indicated that to have experienced non-physical abuse had a significant association with assertiveness to initiate sexual activity, assertiveness to refuse sexual contact, STI prevention, positive attitude towards sexual fantasies, partner-focused dyadic sexual desire, dyadic sexual desire for an attractive person, general sexual desire, sexual excitation, orgasmic capacity, and sexual satisfaction. Moreover, to have experienced physical abuse was significantly associated with the sexual health variables when age, sexual activity, and education level were controlled in women.

## 4. Discussion

The main objective of this study was to compare sexual health variables in men and women who had or had not experienced abuse. To do so, the association that the experience of non-physical abuse and/or physical abuse would have with sexual health dimensions was examined. Both psychosexual variables (sexual assertiveness, erotophilia, and positive attitude towards sexual fantasies) and sexual functioning variables (sexual desire, sexual excitation, orgasmic capacity, erection, and sexual satisfaction) were observed. It was hypothesized that men and women who had experienced non-physical abuse and/or physical abuse would report worse sexual functioning, less sexual assertiveness, and less positive sexual attitude than those who had not experienced abuse.

First, differences across groups about having experienced abuse (Yes/No), either non-physical or physical abuse, have been found in some sociodemographic characteristics. Regarding age, those men who had experienced physical abuse were younger than those who had not experienced abuse, and the mean age of the women who had experienced non-physical or physical abuse was significantly older compared to those who had not experienced abuse. The differences between sexes could be related to gender attitude, where the sexual double standard is notable. The sexual double standard attitude that favors men (i.e., more sexual freedom for men than for women) is associated with experiencing non-physical and physical abuse in women in the partner context [47,58]. This attitude has also been associated with sexual aggression [52,59,60,61] and sexual coercion suffered by women [62]. The results obtained about the distribution of adhesion typologies to the sexual double standard in the Spanish population revealed that the prevalence of the adhesion to woman-favorable typology of sexual double standard lay in younger groups, and for both men and women [63]. Perhaps, as with man-favorable sexual double standard support, the woman-favorable typology is paired with a higher risk of abuse in the partner context. Furthermore, the higher percentage of people who defended this typology in younger groups could be explained by the high probability of young males and older females suffering abuse. The results about sexual activity showed that, although most women informed practicing it, as expected, the percentage of women who have sexual activity was higher in those who had not experienced physical abuse. Finally, concerning the level of education, a higher percentage of men had reported university studies in the group who had not experienced non-physical abuse. For women, in the same direction as men, significant differences have been shown in the level of education between those who had experienced and those who had not experienced abuse. These results are in concordance with Sierra et al. [47], for whom women with higher education levels reported less non-physical abuse than those with primary or secondary education. Thus, a possible interpretation is that people with a higher level of education report fewer abuse experiences. So, education could be one of the most important predictors of IPV [64,65]. Specifically, in the Spanish population, a lower level of education increased accepting IPV and it is a main characteristic to justify the experience of abuse [66,67].

On the one hand, regarding psychosexual variables, the results extend the knowledge about the association between abuse in partnerships and psychosexual variables. Both forms of abuse have been negatively associated with sexual assertiveness in men and women. Those individuals who had experienced non-physical and physical abuse reported lower sexual assertiveness to initiate and to refuse sexual activities. As for preventing STD, this same result was obtained only for men when examining physical abuse experience. These results are consistent with previous studies, which indicate that victimization and abuse experiences could reduce the assertive capacity in sexual contexts [26,27,42]. Differences have been found for both types of abuse in positive attitudes towards sexual fantasies in women, which are less positive in those who have suffered abuse. This finding endorsed the consequence of sexual attitudes as a result of victimization. It is generally known that women report fewer positive attitudes towards sexual fantasies than men [26,28,68], which could explain the fact that this association was found only for women in our study. It should be noted that no association was found between erotophilia and abuse experience in partnerships. This result revealed that, as in other studies [27,28,38,68], attitudes towards sexual fantasies could be a more sensitive variable than the general attitude towards sexuality (i.e., erotophilia) for examining sexual health.

On the other hand, differences in sexual functioning have been found in dyadic sexual desire in both the men and women who had suffered abuse, but not in those who had not experienced it. As expected, partner-focused dyadic sexual desire was lower in both sexes when having suffered physical and non-physical IPV. However, regarding the dyadic sexual desire for an attractive person, men who had experienced physical abuse showed more sexual interest in this type, as well as women who had suffered both forms of abuse. These results support the distinction between both types of dyadic sexual desire (partner-focused and for an attractive person) because they can act independently and differently [52,69,70,71]. It is known that sexual desire plays a basic role in partner relationships [72] and it is an indicator of sexual functioning [71]. Experiencing abuse by the intimate partner affects the partner-focused dyadic desire and it has negative repercussions on the sexual functioning of partner relationships. Furthermore, greater dyadic sexual desire for an attractive person in those who had suffered abuse could indicate that having lived such experiences does not inhibit sexual interest, it could be possible that victims transfer their desire from their partner to other different attractive people. It seems logical that the partner is no longer an effective sexual stimulus as the focus of sexual interest is focused on other people. Regarding solitary sexual desire, men who have experienced physical abuse report more desire for this type. For those men who have experienced physical abuse, this finding endorses the role of sexual desire in partner relationships and sexual functioning by placing more emphasis on solitary sexual behaviors, such as masturbation. The fact that this result is shown only in men can be explained by the sexual differences in this type of desire. Previous studies have pointed out that men report more solitary sexual desire [71], and masturbation more frequently [73,74]. Furthermore, attitudes that favor traditional gender roles, such as the sexual double standard attitude that favors men, could make it difficult for women to experience solitary sexual desire [75,76].

The obtained results indicate that to have experienced abuse affects both men and women’s sexual functioning. It has been shown that gender differences, the experience of abuse, affects more dimensions in women. Previous research with women has emphasized how sexual function is associated with sexual/physical abuse [39]. The abuse experience affects women’s sexual excitation and orgasmic capacity; they are less intense in those women who have suffered both types of abuse, according to previous studies [40,77,78]. This could reveal certain differential aspects between men and women’s subjective orgasm experience [33,79]. As more affected sexual functioning dimensions are found in women, the abuse experienced in the partner context might be more harmful for women. In the present study, the examined percentages of the prevalence of both physical and non-physical abuse in Spain must be interpreted cautiously. As well as the study by Santos-Iglesias et al. [27] with Spanish men, the frequency of behaviors reflecting partner abuse was considered as participants that had suffered abuse were considered who reported from “rarely” to “most of the time”, and participants that had not suffered abuse were only those who reported “never”. Given this strategy for the configuration of abuse/non-abuse groups, high prevalence would be expected.

Finally, this study further evidences the negative association between partner abuse and sexual satisfaction. The relevant impact that both non-physical abuse and physical abuse have on the sexuality of people who have experienced abuse has been shown. Previous studies have observed that the perceived stress in the partner context affects sexual satisfaction [80], and an association between experiencing non-physical and physical abuse with less sexual satisfaction with the abusing partner has been found [23]. A fundamental factor for sexual non-satisfaction could be the partner relationship context itself [81]. Declining sexual satisfaction can be related to relationship problems [82]. Moreover, experiencing abuse could imply emotional intimacy being replaced with more self-protection and controlling the victim [83].

As limitations of the study, despite the large sample, participants were selected by non-probabilistic quota sampling, which limits the generalization of the results to the general population. Furthermore, the study was conducted exclusively with heterosexual participants, so other studies about the experience of abuse in samples with different sexual orientations and with sexual minorities are necessary [84,85,86]. Finally, it would be interesting for future studies to bear in mind the intensity of the abuse suffered, not only examining the dichotomy presence/absence of abuse as in this study.

## 5. Conclusions

In conclusion, the results of this study demonstrate the negative association that the experience of abuse in the partner context has on sexual assertiveness, sexual attitudes, and sexual functioning. Consequences are brought about by both physical and non-physical abuse and in both men and women. However, negative associations are more remarked in women as all the examined sexual functioning dimensions (desire, excitation, orgasm, and sexual satisfaction) are affected. The differences observed in men occur on the dimensions in which subjective components are relevant (i.e., desire and sexual satisfaction); the physiological dimensions (i.e., excitation/erection and orgasm) are not affected. These negative associations could be synthesized in the sexual satisfaction consequence for men and women who have experienced physical and non-physical abuse in the partner context. Future research should consider these findings taking into account biological, physiological, and other psychological dimensions to provide a global perspective about abuse experience.

## Figures and Tables

**Table 1:** Sociodemographic characteristics of men and women and differences between groups of non-physical and physical abuse.

		Men						Women					
		Non-Physical Abuse			Physical Abuse			Non-Physical Abuse			Physical Abuse		
		Yes *n* = 500	No *n* = 323		Yes *n* = 402	No *n* = 500		Yes *n* = 700	No *n* = 716		Yes *n* = 510	No *n* = 700	
				*t*/*χ*^2^			*t*/*χ*^2^			*t*/*χ*^2^			*t*/*χ*^2^
Age (M, SD)		39.14 (13.04)	39.41 (13.47)	−0.29	36.57 (13.34)	40.01 (13)	−3.90 ***	40.22 (13.32)	36.85 (12.96)	4.82 ***	41.30 (13.42)	37.96 (13.04)	4.35 ***
Sexual activity (*n*, %)	Yes	496 (99.20%)	320 (99.10%)	0.04	398 (99%)	496 (99.20%)	0.10	694 (99.10%)	709 (99%)	0.06	502 (98.40%) _a_	697 (99.60%) _b_	4.26 *
	No	4 (0.80%)	3 (0.90%)		4 (1%)	4 (0.80%)		6 (0.90%)	7 (1%)		8 (1.60%) _a_	3 (0.40%) _b_	
Education level (*n*, %)	No studies	7 (1.40%)	4 (1.20%)	9.47 *	8 (2%)	9 (1.80%)	1.92	15 (2.10%)	8 (1.10%)	20.36 ***	19 (3.70%) _a_	10 (1.40%) _b_	11.79 **
	Primary school	99 (19.80%)	53 (16.40%)		64 (15.90%)	85 (17%)		147 (21%) _a_	105 (14.70%) _b_		111 (21.80%)	123 (17.60%)	
	High school	170 (34%) _a_	86 (26.60%) _b_		121 (30.10%)	168 (33.60%)		193 (27.60%)	170 (23.70%)		132 (25.90%)	179 (25.60%)	
	University	224 (44.80%) _a_	180 (55.70%) _b_		209 (52%)	238 (47.60%)		345 (49.30%) _a_	433 (60.50%) _b_		248 (48.60%) _a_	388 (55.40%) _b_	

Note: Sexual activity: have sexual relations within the partner’s relationship. No studies: have not completed primary school. M = mean; SD = standard deviation; *t* = Student’s; *χ*^2^ = Chi-square; *** *p* < 0.001; ** *p* < 0.01, * *p* < 0.05. Different subscript letters denote the proportions of groups that significantly differ.

**Table 2:** Differences of each type of abuse on the sexual health variables controlling covariates in men.

	Non-Physical Abuse					Physical Abuse				
	Yes	No				Yes	No			
	M (SD)	M (SD)	F	*p*	η^2^	M (SD)	M (SD)	F	*p*	η^2^
Assertiveness to initiate sexual activity	13.92 (4.31)	15.23 (4.80)	15.22	<0.001	0.018	13.89 (4.07)	14.70 (4.31)	9.73	0.002	0.011
Assertiveness to refuse sexual contact	11.04 (5.41)	12.82 (6.19)	17.16	<0.001	0.020	10.99 (5.08)	11.80 (5.68)	7.32	0.007	0.008
STI prevention	12.68 (7.57)	13.65 (7.36)	2.24	0.135		12.61 (6.78)	13.19 (7.63)	5.25	0.022	0.006
Erotophilia	34.07 (6.54)	34.18 (7.32)	0.011	0.917		34.22 (6.39)	34.51 (6.57)	2.33	0.127	
Sexual fantasies	30.22 (6.95)	31.21 (7.92)	2.84	0.092		30.87 (6.54)	30.93 (7.44)	1.25	0.263	
Partner-focused dyadic sexual desire	41.43 (7.40)	42.77 (7.43)	5.63	0.018	0.007	41.45 (7.07)	42.11 (7.46)	7.59	0.006	0.008
Dyadic sexual desire for an attractive person	8.75 (4.58)	8.21 (4.73)	2.83	0.093		9.29 (4.18)	8.46 (4.44)	8.84	0.003	0.010
Solitary sexual desire	15.61(7.56)	15.63 (8.63)	0.010	0.930		17.10 (7.16)	15.34 (7.85)	8.64	0.003	0.010
General sexual desire	3.63 (1.0)	3.84 (0.85)	9.28	0.002	0.011	3.65 (0.98)	3.71 (0.98)	3.15	0.077	
Sexual excitation	3.65 (0.88)	3.78 (0.83)	3.80	0.052		3.64 (0.95)	3.70 (0.94)	3.10	0.079	
Orgasmic capacity	3.71 (0.88)	3.79 (0.80)	1.72	0.190		3.78 (0.87)	3.74 (0.91)	0.00	0.964	
Erection	3.77 (0.80)	3.85 (0.74)	2.23	0.136		3.84 (0.77)	3.84 (0.80)	1.53	0.217	
Sexual satisfaction	3.50 (1.06)	3.81 (0.95)	17.90	<0.001	0.021	3.52 (1.10)	3.65 (1.01)	5.55	0.019	0.006

Note: Covariates were education level for non-physical abuse analyses, and age for physical abuse analyses. η^2^ = partial eta squared. STI = Sexually Transmitted Infections.

**Table 3:** Differences of each type of abuse on the sexual health variables controlling covariates in women.

	Non-Physical Abuse					Physical Abuse				
	Yes	No				Yes	No			
	M (SD)	M (SD)	F	*p*	η^2^	M (SD)	M (SD)	F	*p*	η^2^
Assertiveness to initiate sexual activity	13.04 (5.31)	14.83 (5.27)	23.69	<0.001	0.016	12.44 (5.12)	14.28 (5.38)	21.79	<0.001	0.018
Assertiveness to refuse sexual contact	14.65 (4.74)	16.78 (5.15)	45.74	<0.001	0.031	13.48 (4.57)	16.31 (5.12)	80.25	<0.001	0.062
STI prevention	16.15 (6.97)	18.00 (6.86)	11.47	0.001	0.008	15.02 (7.24)	17.81 (6.75)	33.01	<0.001	0.027
Erotophilia	31.60 (7.83)	33.06 (7.70)	2.00	0.157		30.96 (8.01)	32.55 (7.87)	2.94	0.087	
Sexual fantasies	25.57 (8.60)	27.91 (9.27)	8.55	0.004	0.006	24.72 (8.75)	27.30 (9.18)	10.33	0.001	0.009
Partner-focused dyadic sexual desire	37.24 (9.88)	40.08 (9.13)	13.35	<0.001	0.009	35.72 (10.72)	39.32 (9.26)	20.79	<0.001	0.017
Dyadic sexual desire for an attractive person	6.19 (4.72)	5.99 (4.60)	4.80	0.029	0.003	6.83 (4.65)	5.87 (4.53)	23.60	<0.001	0.019
Solitary sexual desire	10.90 (8.32)	11.77 (9.11)	1.07	0.300		11.55 (8.35)	11.17 (8.98)	3.08	0.080	
General sexual desire	3.17 (1.38)	3.56 (1.13)	19.48	<0.001	0.014	3.03 (1.44)	3.40 (1.25)	10.83	0.001	0.009
Sexual excitation	3.19 (1.30)	3.56 (1.06)	20.05	<0.001	0.014	3.07 (1.32)	3.43 (1.19)	11.57	0.001	0.010
Orgasmic capacity	3.23 (1.31)	3.63 (1.09)	22.08	<0.001	0.015	3.12 (1.37)	3.46 (1.22)	9.18	0.003	0.008
Sexual satisfaction	3.27 (1.37)	3.69 (1.09)	28.19	<0.001	0.020	3.15 (1.39)	3.55 (1.26)	16.51	<0.001	0.014

Note: Covariates were age and education level for non-physical abuse analyses, and age, sexual activity, and education level for physical abuse analyses. η^2^ = partial eta squared. STI = Sexually Trasmitted Infections.

## Data Availability

The data presented in this study are available on request from the corresponding author. The data are not publicly available due to privacy.
